# Prognostic Value of GPNMB, EGFR, p-PI3K, and Ki-67 in Patients with Esophageal Squamous Cell Carcinoma

**DOI:** 10.1155/2022/9303081

**Published:** 2022-08-31

**Authors:** Bo Wang, Mengyan Li, Anna Su, Yongmei Gao, Yan Shi, Chao Li, Wenying Liu, Liping Su, Wan Li, Yuqing Ma

**Affiliations:** ^1^Department of Pathology, The First Affiliated Hospital, Xinjiang Medical University, Urumqi, Xinjiang, China; ^2^Xinjiang Medical University, Urumqi, Xinjiang, China; ^3^Department of Pathology, Gem Flower Hospital, Changji, Xinjiang, China; ^4^Internal Medicine, Urumqi First People's Hospital, Urumqi, Xinjiang, China; ^5^Department of Pathology, Baoji Traditional Chinese Medicine Hospital, Baoji, Xian, China; ^6^Department of RICU, The First Affiliated Hospital, Xinjiang Medical University, Urumqi, Xinjiang, China; ^7^National Cancer Center/National Cancer Clinical Medical Research Center/Cancer Hospital, Hebei Chinese Academy of Medical Sciences, Langfang, Hebei, China

## Abstract

**Background:**

GPNMB is a newly discovered tumour-promoting factor that may promote tumour cell progression by activating the PI3K/AKT pathway by EGFR. However, there are insufficient studies about GPNMB in ESCC. This study investigated the relationship between GPNMB and EGFR/PI3K pathway genes in ESCC.

**Methods:**

The expression levels of GPNMB, EGFR, p-PI3K, and Ki-67 were examined using immunohistochemistry. Statistical analysis was done by SPSS 22.0 and R.

**Results:**

GPNMB mRNA expression is higher in ESCC compared with paracancerous tissues. The expression of EGFR, PIK3CA, PIK3CB, and AKT1 was increased in GPNMB upregulated samples. GPNMB expression was positively correlated with EGFR, p-PI3K, and Ki-67 expression. GPNMB was expressed higher in the AJCC III stage, lymph node metastasis, and moderately poorly differentiated patients. EGFR was higher expressed in patients with vascular invasion; p-PI3K expression in Kazak was higher than that in Han; Ki-67 expression was higher in tumour size ≥ 3 cm. Patients with high expression of GPNMB, p-PI3K, and Ki-67 had worse OS. p-PI3K, Ki-67, nerve invasion, and lymphatic metastasis were independent risk factors, and postoperative adjuvant therapy was a protective factor in ESCC.

**Conclusion:**

As a tumour-promoting factor, GPNMB is expected to be a potential target for ESCC.

## 1. Introduction

Esophageal cancer is a common malignant tumour in the digestive system with an inferior prognosis [1, 2], divided into two subtypes: esophageal squamous cell carcinoma (ESCC) and esophageal adenocarcinoma. ESCC is mainly prevalent in Asian populations [3], and Xinjiang is a high-incidence area of ESCC. Studies have shown that the incidence and mortality of ESCC in Kazak are higher in Han [4, 5]. Currently, the main treatment method for ESCC is still surgery combined with postoperative adjuvant chemoradiotherapy, which lacks targeted therapy [6, 7]. Accurate classification of patients and targeted therapy based on biomarkers are effective ways to improve prognosis [8, 9].

Previously, our team performed an ITRAQ proteomic analysis of ESCC and normal esophageal tissue [10]. We found that the glycoprotein nonmetastatic melanoma protein B (GPNMB) is one of the proteins significantly upregulated in ESCC. Other studies have also shown that GPNMB is highly expressed in various malignancies and is closely related to prognosis [11–13], but its relationship with ESCC is unclear.

GPNMB is a type I transmembrane protein consisting of 576 amino acids and contains three parts: an extracellular domain, a transmembrane region, and an intracellular domain [14]. The GPNMB gene is located on chromosome 7q15 and is involved in cell proliferation, apoptosis, and differentiation [15, 16]. Interestingly, the hem immunoreceptor tyrosine activation motif in the intracellular domain of GPNMB has tyrosine kinase activity [17], enabling it to bind to neighbouring receptor protein tyrosine kinases (RPTKs) and activate downstream signalling pathways, which may be the primary molecular mechanisms of GPNMB in tumour progression. Epidermal growth factor receptor (EGFR) is the most common RPTK, which can form dimers with itself or other receptors to activate downstream signalling pathways leading to phosphorylation cascades [18]. Phosphatidylinositol 3-kinase (PI3K) is a downstream protein of EGFR that can be phosphorylated by EGFR and converted from phosphatidylinositol 4,5-bisphosphate PIP2 to phosphatidylinositol 3,4,5-triphosphate PIP3. The phosphorylation process marks the activation of the EGFR/PI3K signalling pathway, and the abnormal activation of this pathway plays a key role in the occurrence and development of tumours [19].

Recently, some scholars have explored the relationship between the GPNMB and EGFR/PI3K signalling pathways. Lin et al. found that GPNMB activates the downstream tyrosine kinase signalling pathway by forming a heterodimer with EGFR [20]. Jin et al. effectively inhibited the PI3K/AKT pathway by blocking GPNMB, thereby reducing the proliferation and metastasis of osteosarcoma cells [14]. Ki-67 is a typical cell proliferation marker, significantly associated with the poor prognosis of various malignant tumours [21, 22]. It has been used in clinical practice as an important marker for refining breast cancer grading and guiding treatment. Recent studies have found that Ki-67 can reflect the activation level of the PI3K/AKT signalling pathway [23].

In summary, the purpose of this study was to preliminarily analyze the expressions of GPNMB, EGFR, p-PI3K, and Ki-67 in ESCC and their relationship with clinicopathological parameters and the effects of the expressions of the four proteins on the prognosis of ESCC.

## 2. Materials and Method

### 2.1. Patient and Tissue Samples

All patients gave informed consent before sample collection, and the Ethics Committee approved this study at the First Affiliated Hospital of Xinjiang Medical University (20180223-08). 240 ESCC paraffin-embedded samples and paired adjacent noncancerous tissues between January 2012 and December 2018 were collected and fabricated into tissue chips. Follow-up was conducted by inquiring about medical records and telephone follow-up until December 2020. Inclusion criteria were as follows: (1) patients with ESCC; (2) patients who did not receive radiotherapy or chemotherapy before surgery; (3) the esophagus was the primary lesion site; and (4) patients of Han or Kazak ethnicity. The exclusion criteria included the following: (1) patients with adenocarcinoma of the esophagus; (2) patients who received radiotherapy or chemotherapy before surgery; (3) patients with tumour metastasis to the esophagus; (4) other ethnic groups, including Uyghurs, Mongolians, etc.; (5) patients who died during the operation and in hospital; (6) patients with Tis and T1a stage; and (7) patients with incomplete tissue specimens. According to the above criteria, 6 cases of preoperative chemotherapy, 3 cases of T1a stage, and 5 cases of sparse tissue or specimen detachment were excluded from the 240 samples. 2 deputy chief pathologists assessed the final included 226 specimens to confirm the histological diagnosis and differentiation of ESCC. The following information was recorded for each patient: age, sex, nationality, tumour size, location, differentiation, depth of invasion, AJCC stage (according to the 2017 eighth edition of AJCC), lymph node metastasis, vascular invasion, neural invasion, and postoperative adjuvant therapy. Information on these variables is recorded in Table 1.

RNA-seq expression data of 81 ESCC tissues and 11 paracancerous tissues were collected from the TCGA database (https://portal.gdc.cancer.gov/). Another ESCC dataset, GSE161533, with 28 matched ESCC and standard esophageal samples, was obtained from the GEO database (https://www.ncbi.nlm.nih.gov/geo/query/acc.cgi?acc=GSE161533).

### 2.2. Antibodies and Reagents

The primary antibodies and reagents were GPNMB, EGFR, p-PI3K, and Ki-67. Other reagents were endogenous peroxidase blocker, goat serum working solution, enzyme-labelled goat antirabbit IgG polymer, and 2-amino-benzidine (DAB). Anti-GPNMB antibody and anti-Ki-67 antibody were purchased from Abcam, UK; anti-EGFR antibody and anti-p-PI3K antibody were purchased from Affinity Biosciences, USA; and other reagents were purchased from the Zhongshan Jinqiao Company, China.

### 2.3. Immunohistochemistry (IHC)

The tissue chips were sliced into 4 *μ*m sections, deparaffinized in xylene, and rehydrated in 100, 95, 80, and 70% ethanol. Following treatment with 3% hydrogen peroxide to block endogenous peroxidase activity, the sections were heated with EDTA (pH 9.0) in boiling water at 100°C for antigen retrieval. The sections were then treated with goat serum (ZSGB-BIO, ZLI-9022) at room temperature to block nonspecific antigens for 30 min. Then, use anti-GPNMB antibody (1 : 1000, Abcam, AB222109), anti-EGFR antibody (1 : 200, Affinity, AF604), anti-p-PI3K antibody (1 : 200, Affinity, AF3241), and anti-Ki-67 antibody (1 : 200, Abcam, AB15580) overnight at 4°C. Sections were incubated with a peroxide-labelled polymer (ZSGB-BIO, PV-6001) as a secondary antibody for 30 min. The slides were subsequently stained with DAB, dehydrated, sealed, and observed under a light microscope (DM300; Leica Microsystems GmbH; magnifications, ×10 and ×40).

### 2.4. IHC Score

Image-Pro Plus (version 6.0 for Windows) was used to identify IHC images. The H-score was calculated by multiplying the percentage of positive cells by the weighted intensity of staining [24–26]. The total staining intensity and the ratio of positive and negative squamous and stromal cells in the captured fields were counted at 40x magnification, and semiquantitative H-scores were obtained for each field. The H-score was obtained by applying the following formula: H − score = 1 × (%weak staining) + 2 × (%medium staining) + 3 × (%strong staining) [26, 27]. GPNBM, EGFR, and p-PI3K scores ranged from 0 to 300. On the other hand, Ki-67 showed no significant difference in staining intensity, only the percentage of positive cells was scored, and the score ranged from 0 to 100 [25].

### 2.5. Statistical Analysis

All statistical analyses were performed using SPSS 22.0 (SPSS, Chicago, IL) and R (Version.4.0.2). Paired samples were tested by paired *T* test. The rank-sum test was used to compare the data differences among groups, the Wilcoxon rank-sum test was used for the comparison between two samples, and the Kruskal-Wallis rank-sum test was used for the comparison between multiple samples. Pearson was used to analyze the correlation among GPNMB, EGFR, p-PI3K, and Ki-67. Survival curves were drawn using the Kaplan-Meier method, and the log-rank test was used for comparison. Univariate Cox analysis was used to screen variables with prognostic significance, and variables with *P* < 0.05 were selected for multivariate analysis. The Cox proportional hazard model was used for stepwise regression and for screening variables with independent prognostic significance. *P* < 0.05 was considered as statistically significant.

## 3. Result

### 3.1. The Expression of GPNMB Was Increased in ESCC

**Figure 1:**
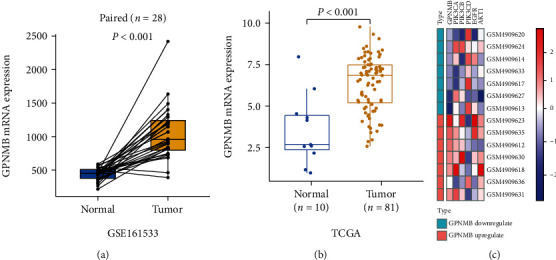
GPNMB mRNA expression was increased in ESCC and correlated with the PI3K pathway. (a) The GPNMB expression difference between 28 pairs of ESCC and paired paracancer tissues in the GEO database was analyzed by paired *T*-test. Results are presented as means ± SD; the box plot shows the median value (quartile) of the two groups. (b) Differences in GPNMB expression between ESCC (*n* = 81) and normal esophageal tissues (*n* = 10) in TCGA were analyzed by Wilcoxon rank-sum test. The results were counted as the median difference; the box plot shows the median value (quartile) of the two groups. (c) In the GEO (GSE161533) database, ESCC samples with the highest (*n* = 7) and lowest (*n* = 7) GPNMB expression were selected for differential gene analysis. Heat maps of PI3K pathway-related gene expression in GPNMB upregulated and downregulated samples. Red indicates upregulated genes and blue indicates downregulated genes.

**Figure 2:**
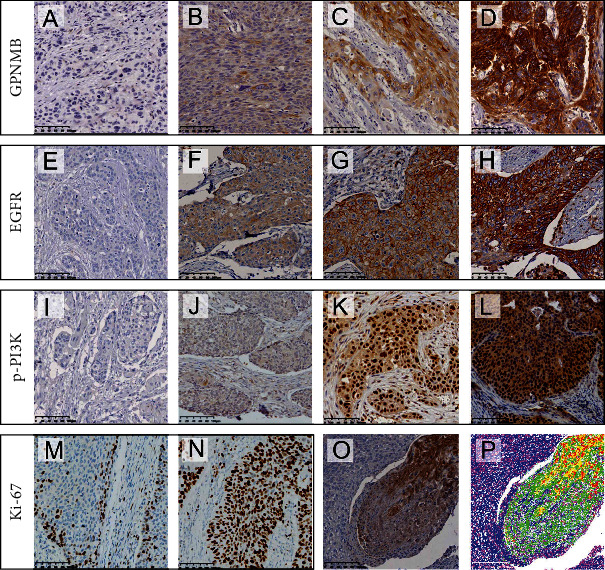
IHC of GPNMB, EGFR, p-PI3K, and Ki-67 in ESCC. (a) to (d) represent colourless, weak, medium, and strong colours of GPNMB, respectively; (e) to (h) represent colourless, weak, medium, and strong colours of EGFR, respectively; (i) to (l) represent colourless, weak, medium, and strong colours of p-PI3K, respectively; (m) and (n) represent the images of low and high expressions of Ki-67, respectively; (p) represents the image after Image-Pro Plus processing (red indicates the range of strong staining, yellow indicates the moderately stained range, and green indicates the weakly stained range); (o) represents the original image.

The mRNA data of 28 pairs of ESCC and paracancer tissues in the GEO database (GSE161533 dataset) were analyzed. The results showed that the expression of GPNMB in ESCC tissues was significantly higher than that in the paired adjacent tissues (*P* < 0.001, Figure 1(a)). The expression of GPNMB in the TCGA database was also higher in ESCC samples (*P* < 0.001, Figure 1(b)). IHC results showed that GPNMB was hardly expressed in the esophageal squamous epithelium (Supplementary Figures A, B) but was stained in 96.46% (218/226) of ESCC tissues, mainly expressed in the cell membrane and cytoplasm. According to the staining intensity, it was divided into colourless, weak colour, medium colour, and strong colour (Figures 2(a)–2(d)).

### 3.2. GPNMB Expression Was Associated with the EGFR/PI3K Pathway

**Figure 3:**
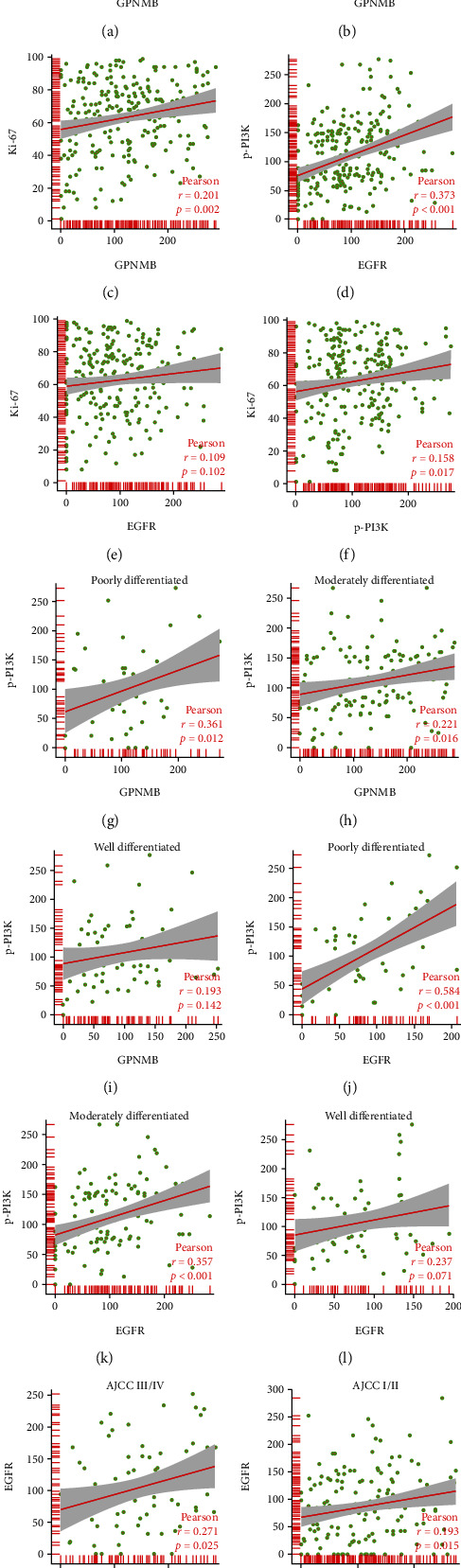
Correlation between GPNMB, EGFR, p-PI3K, and Ki-67. Pearson was used to analyze the correlation between (a) GPNMB and EGFR, (b) GPNMB and p-PI3K, (c) GPNMB and Ki-67, (d) EGFR and p-PI3K, (e) EGFR and Ki-67, (f) p-PI3K and Ki-67, (g–i) GPNMB and EGFR at different differentiation levels, (j–l) EGFR and p-PI3K at different differentiation levels, and (m, n) GPNMB and EGFR at different AJCC stages.

In the GEO (GSE161533) database, ESCC samples with the highest (*n* = 7) and lowest (*n* = 7) GPNMB expressions were selected for differential gene analyses. The results showed that PIK3CA and PIK3CB, as genes encoding PI3K proteins, were increased in the GPNMB upregulated group; EGFR and AKT1, as upstream and downstream of PI3K, were also highly expressed in the GPNMB upregulated group (Figure 1(c)). IHC results showed that EGFR was expressed in ESCC and normal esophageal tissues, mainly in the cell membrane and cytoplasm. The expression of the normal squamous epithelium was shown in Supplementary Figures C and D, while colourless, weak, medium, and strong colours in ESCC are shown in Figures 2(e)–2(h). p-PI3K was expressed in the cytoplasm and nucleus of ESCC and normal esophageal tissue. It was expressed in the normal squamous epithelium as shown (Supplementary Figures E, F) and in ESCC tissue according to colourless, weak, medium, and strong colours displayed (Figures 2(i)–2(l)). Ki-67 was expressed in the nuclei of ESCC and normal tissues, expressed in basal cells in the normal squamous epithelium (Supplementary Figures G, H), and was widely expressed in tumour cells in ESCC. As their staining intensity was not significantly different, we used a percent staining score [25] for their assessment, with low and high scores as shown in Figures 2(m) and 2(n). The staining scoring system of Image-Pro Plu software for pictures is shown in Figures 2(o) and 2(p). Correlation analysis showed that GPNMB was correlated with EGFR (*R* = 0.238, *P* < 0.001), p-PI3K (*R* = 0.230, *P* < 0.001), and Ki-67 (*R* = 0.201, *P* = 0.002) and p-PI3K was positively correlated with EGFR (*R* = 0.373, *P* < 0.001) and Ki-67 (*R* = 0.158, *P* = 0.017), while EGFR was not correlated with Ki-67 (Figures 3(a)–3(f)). We also found a stronger correlation between GPNMB and p-PI3K in poorly differentiated patients (*R* = 0.361, *P* = 0.012; Figure 3(g)), whereas they were less or not correlated in moderately and well-differentiated patients (Figures 3(h) and 3(i)). Likewise, the association of EGFR with p-PI3K was more pronounced in poorly differentiated patients than in moderately and well-differentiated patients (Figures 3(j)–3(l)). The association of GPNMB with EGFR was also higher in III/IV stage than in I/II stage (*R* = 0.271, *P* = 0.025 vs. *R* = 0.193, *P* = 0.015, respectively; Figures 3(m) and 3(n)).

### 3.3. The Relationship between GPNMB, EGFR, p-PI3K, and Ki-67 with Clinicopathology

**Figure 4:**
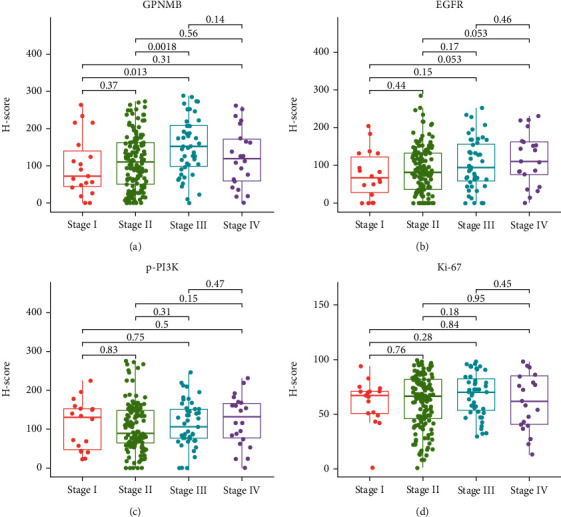
Expression of four proteins at different AJCC stages. The Kruskal-Wallis rank-sum test was used to analyze the expression differences of (a) GPNMB, (b) EGFR, (c) p-PI3K, and (d) Ki-67 in different stages. The box plot shows the median value (quartile) of the two groups.

**Figure 5:**
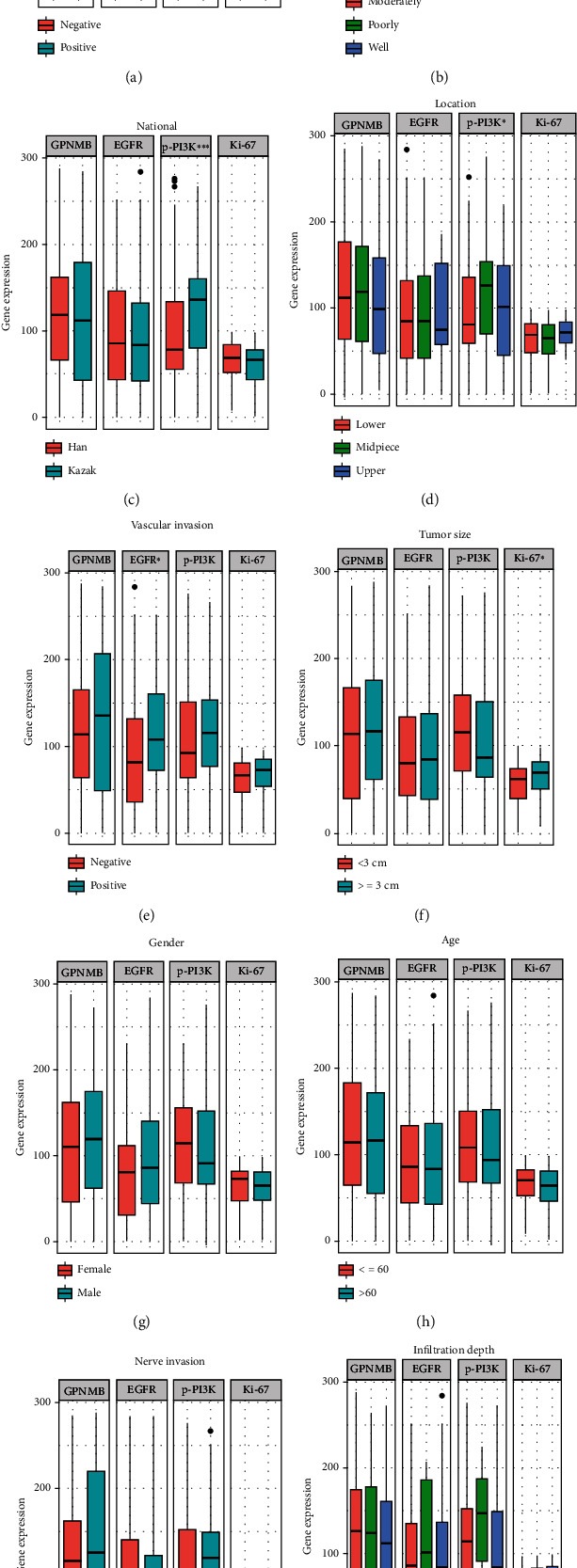
Expression of GPNMB, EGFR, p-PI3K, and Ki-67 in different clinicopathological stratifications. The expression difference of the 4 proteins between (a) different lymph node metastasis states, (b) different differentiation levels, (c) different nationalities, (d) different ESCC locations, (e) vascular invasion or not, (f) different tumour sizes, (g) different genders, (h) different age groups, (i) nerve invasion or not, and (j) different infiltration depths. The Wilcoxon rank-sum test was used for the comparison between two samples, and the Kruskal-Wallis rank-sum test was used for the comparison between multiple samples. Adjusted *P* values are shown as follows: ns: not significant, ^∗^*P* < 0.05, ^∗∗^*P* < 0.01, and ^∗∗∗^*P* < 0.001.

The expression of GPNMB was higher in AJCC stage III than in stage I (*P* = 0.013) and stage II (*P* = 0.0018, Figure 4(a)). The expression of EGFR increased with the stage, but there was no statistical difference between different stages (Figure 4(b)). The expression of p-PI3K and Ki-67 did not differ significantly between different stages (Figures 4(c) and 4(d)). GPNMB expression was higher in the lymph node metastasis group than in the nonmetastasis group (*P* <0.001, Figure 5(a)). GPNMB expression was highest in moderately differentiated and lowest in well-differentiated (*P* < 0.01, Figure 5(b)). The expression of p-PI3K was higher in Kazak patients (*P* < 0.001, Figure 5(c)). Differences in p-PI3K were also reflected in different locations, with the highest in the middle ESCC and the lowest in the upper ESCC (*P* < 0.05, Figure 5(d)). In patients with vascular invasion, the expression of all four proteins was higher than that in patients without vascular invasion (Figure 5(e)), among which EGFR expression was statistically significant (*P* < 0.05). The expression of Ki-67 was higher in patients with tumour size ≥ 3 cm (*P* < 0.05, Figure 5(f)). There were no statistically significant differences in the expression of the four proteins among groups of different gender, age, neural invasion, and depth of invasion (Figures 5(g)–5(j)).

### 3.4. The Relationship between GPNMB, EGFR, p-PI3K, and Ki-67 with the Prognosis of ESCC

**Figure 6:**
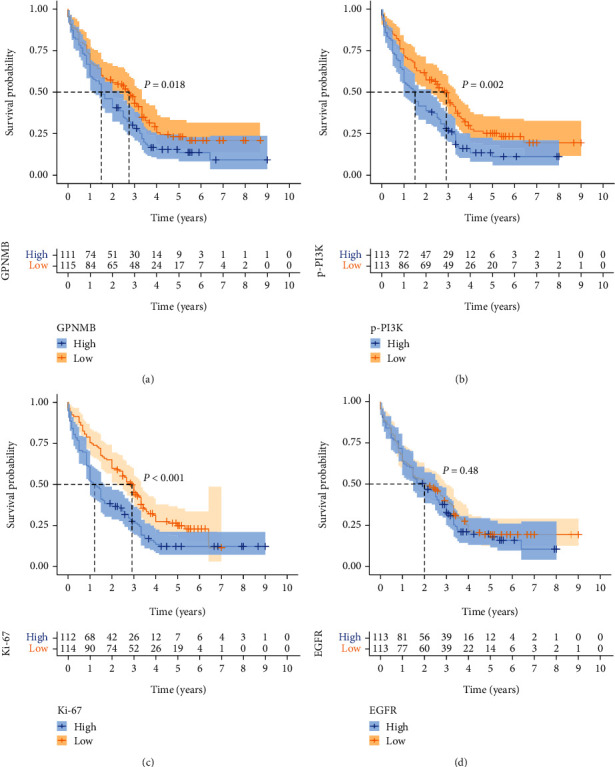
Kaplan–Meier survival curves for patients. The difference in prognosis between the high and low expression groups of (a) GPNMB, (b) p-PI3K, (c) Ki-67, and (d) EGFR. Log-rank test was used to compare the difference between two curves. The pale area around the curve was the 95% confidence interval.

**Figure 7:**
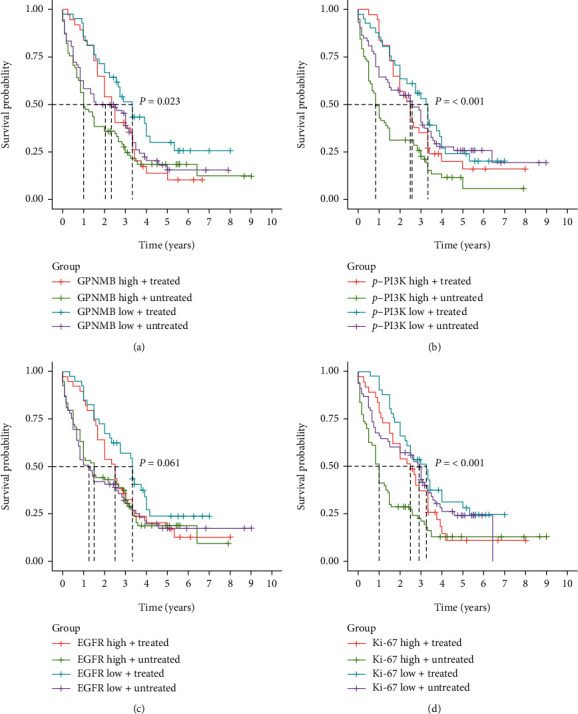
Effects of GPNMB, EGFR, p-PI3K, and Ki-67 combined with adjuvant therapy on prognosis. (a) The prognostic difference of GPNMB expression with or without combined treatment; (b) the prognostic difference of p-PI3K expression with or without combined treatment; (c) the prognostic difference of EGFR expression with or without combined treatment; (d) the prognostic difference of Ki-67 expression with or without combined treatment. Log-rank test was used to compare the difference of multiple curves.

The four proteins were divided into high and low expression groups using the median as a cut-off, and a prognostic (OS) analysis was performed. Patients with low expression of GPNMB (*P* = 0.018), p-PI3K (*P* = 0.002), and Ki-67 (*P* < 0.001) had better prognosis (Figures 6(a)–6(c)), whereas EGFR expression was not associated with prognosis (Figure 6(d)). Subsequently, patients were stratified according to whether they received postoperative adjuvant therapy, and the effects of the expression of the four proteins on treatment were explored. The GPNMB low expression+treatment group had the best prognosis, with a median survival time of 3.3 years (Figure 7(a)). The median survival time of the p-PI3K high expression+treatment group was close to that of the p-PI3K low expression group. It was significantly better than the p-PI3K high expression untreated group (Figure 7(b)). The EGFR high expression+treatment group had a longer median survival than the high expression untreated group, but the difference was not statistically significant (Figure 7(c)). The median survival time of the untreated group with high expression of Ki-67 was only one year. In contrast, the median survival time of the high expression+treatment group was longer and was similar to that of the low expression group, and the difference was statistically significant (Figure 7(d)).

### 3.5. Univariate and Multivariate Analysis of ESCC

**Figure 8:**
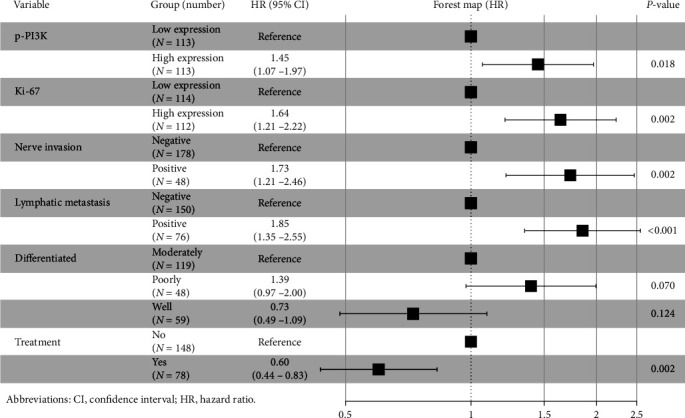
Multivariate analysis and forest maps. High expression of p-PI3K, high expression of Ki-67, nerve invasion, and lymphatic metastasis were independent risk factors for ESCC patients; treatment was an independent protective factor in ESCC patients.

Independent prognostic factors of ESCC were screened by univariate and multivariate Cox analyses. Univariate Cox showed that p-PI3K (*P* < 0.001), Ki-67 (*P* < 0.001), lymphatic metastasis (*P* < 0.001), differentiated (*P* < 0.001), nerve invasion (*P* = 0.004), treatment (*P* = 0.018), and AJCC (*P* < 0.001) were prognostic factors for ESCC, while GPNMB (*P* = 0.056), EGFR (*P* = 0.466), tumour size (*P* = 0.127), location (*P* = 0.760), infiltration depth (*P* = 0.275), vascular invasion (*P* = 0.064), age (*P* = 0.887), gender (*P* = 0.096), and nationality (*P* = 0.938) were not statistically significant (Table 2). With *P* < 0.05 as the inclusion criterion, p-PI3K, Ki-67, lymphatic metastasis, differentiation, nerve invasion, treatment, and AJCC were included in the multivariate analysis. The results showed that high expression of p-PI3K (*P* = 0.018), high expression of Ki-67 (*P* = 0.002), nerve invasion (*P* = 0.002), and lymphatic metastasis (*P* < 0.001) were independent risk factors for ESCC patients; treatment (*P* = 0.002) was an independent protective factor in ESCC patients (Figure 8).

## 4. Discussion

In China, 90% of EC patients have developed to the middle and late stages. Surgery is the primary method for the treatment of EC, but the long-term efficacy is poor, and the overall 5-year survival rate is less than 20%. With the development of research, the application of molecular targeted drugs and immunotherapy has brought new hope to patients with advanced EC. The search for new molecular therapeutic targets is significant for treating EC.

GPNMB can promote tumorigenesis and development in various malignant tumours as a newly discovered transmembrane protein. Maric et al. found that GPNMB enhances vascular endothelial growth factor signal transduction in breast cancer cells through NRP-1 expression and activates the tyrosine kinase signalling pathway in an RGD motif-dependent manner to promote tumour progression [28]. In the study of glioma, Bao et al. found that GPNMB promotes tumour proliferation and metastasis by regulating matrix metalloproteinases through the Wnt/*β*-catenin pathway [29]. Similarly, the researchers attenuated the proliferation and migration of bladder cancer cells through GPNMB gene knockout while reducing the expression of MMP-2, MMP-9, and *β*-catenin and demonstrated that high GPNMB expression is a risk factor for bladder cancer [30]. Our results showed that the expression of GPNMB in ESCC tissues was higher than that in adjacent tissues; the expression rate of GPNMB in primary ESCC patients was 96.5% (218/226); the expression of GPNMB was correlated with the AJCC stage, lymph node metastasis, and degree of differentiation. The overall survival of patients with high GPNMB expression was significantly shorter than those with low GPNMB expression. Therefore, GPNMB may serve as a novel tumour therapeutic target and prognostic marker of ESCC.

Recently, some scholars have studied the relationship between the GPNMB and EGFR/PI3K signalling pathways. Lin et al. found that GPNMB activates the downstream tyrosine kinase pathway by forming a heterodimer with EGFR [20]. Han et al. found that GPNMB can bind to the C-terminus of EGFR, assist in the phosphorylation of Y845, activate mutated EGFR in a ligand-independent manner, and then open the downstream signalling pathway [31]. EGFR belongs to the ErbB family, which includes HER1 (erbB1, EGFR), HER2 (erbB2, NEU), HER3 (erbB3), and HER4 (erbB4). EGFR activates downstream signalling pathways by dimerizing with itself or other receptors, causing phosphorylation cascades and promoting aberrant activation of downstream kinase pathways [18]. There are two main pathways for downstream signal transduction of EGFR: Ras/Raf/MEK/ERK-MAPK and PI3K/Akt/mTOR. When the PI3K/AKT/mTOR pathway is abnormally activated, it promotes the occurrence and development of various cancers, including EC [32–34]. Shang et al. found that high expression of PI3K-p85*α*, EGFR, and p53 in ESCC was significantly associated with poor prognosis; multivariate Cox regression analysis demonstrated that the combination of the three proteins was an independent prognostic factor in ESCC patients [35]. As an indicator of cell proliferation, Ki-67 can effectively assess the prognosis of various malignancies, including ESCC [21, 22]. The study of Zhou et al. showed that the expression level of Ki-67 can reflect the activation level of the PI3K/AKT signalling pathway [23]. Therefore, we hypothesized that GPNMB may be related to the expression of EGFR/PI3K pathway proteins and Ki-67 and that they act together in ESCC, but there is still a lack of relevant research.

We further performed IHC on EGFR, p-PI3K, and Ki-67 to analyze their relationship with GPNMB and clinicopathological factors and their prognostic significance. The results showed that EGFR was expressed in the cell membrane and cytoplasm, and its high expression was associated with vascular invasion (*P* < 0.05). p-P3IK was expressed in the cytoplasm and nucleus, and its expression correlated with nationality (*P* < 0.001) and tumour location (*P* < 0.05), and patients with high expression had a worse prognosis (*P* = 0.002). Ki-67 was expressed in the nucleus, and its expression correlated with tumour size (*P* < 0.05). Patients with a high expression of Ki-67 had shorter overall survival (*P* < 0.001). We performed a correlation analysis to explore the relationship between GPNMB and EGFR, p-PI3K, and Ki-67. The correlation between GPNMB and EGFR was lower (*R* = 0.238, *P* < 0.001), but we found a higher correlation between the two in AJCC III-IV patients than in AJCC I-II patients (*R* = 0.271, *P* = 0.025 and *R* = 0.193, *P* = 0.015, respectively; Figures 3(m) and 3(n)). GPNMB was also associated with p-PI3K (*R* = 0.230, *P* < 0.001) and Ki-67 (*R* = 0.201, *P* = 0.002), and GPNMB was more significantly associated with p-PI3K in poorly differentiated patients (*R* = 0.361, *P* = 0.012), with little or no correlation in moderately and well-differentiated patients. Similarly, the association between EGFR and p-PI3K was most significant in poorly differentiated patients (*R* = 0.584, *P* < 0.001). The above results suggest that GPNMB was associated with EGFR and p-PI3K in ESCC, and with tumour progression, the correlation between them became higher and higher.

To further explore the influence of the four proteins on clinical treatment, the prognosis of patients was evaluated in combination with postoperative adjuvant therapy. The survival curves of GPNMB and p-PI3K have similar characteristics. Patients with high expression have a poor prognosis without postoperative adjuvant therapy. Interestingly, after treatment, the 1-year survival rate of patients with high expression was similar to that of patients with low expression; however, after one year, the difference in survival between the two groups gradually increased (Figures 6(a) and 6(b)). The results suggest that the high expression of GPNMB and p-PI3K may be the reason for the resistance of ESCC to adjuvant therapy. The drug resistance mechanism of PI3K to tumours has been confirmed in previous studies. Jin et al. found that the expression of PI3K/AKT signalling pathway genes was upregulated in drug-resistant small cell lung cancer, and the use of PI3K inhibitors enhanced the sensitivity of chemotherapeutic drugs [36]. Gris-Oliver et al. demonstrated that PI3K pathway activation induces resistance to eribulin in HER2-breast cancer patients, while PI3K inhibits apoptosis and reduces drug efficacy by promoting P21 [37]. In contrast, there are few studies on GPNMB in tumour resistance. The drug resistance model constructed by Sun et al. showed that 12 prognostic features, including GPNMB, could predict glioma patients' resistance or susceptibility to targeted therapy [38]. Xu et al. studied mouse colon adenocarcinoma cells (MC38). They found that GPNMB was significantly upregulated in PD-1-resistant tumour cells. Deleting GPNMB in drug-resistant cells successfully restored tumour sensitivity to anti-PD-1 therapy. It is thought that GPNMB may be a marker of immunotherapy resistance [39]. These studies are consistent with our results that GPNMB, a potential therapeutic and drug resistance target in ESCC, may be an underestimated marker in tumour research. More in-depth studies are needed to explore its value. Surprisingly, the survival curves of treated patients with high Ki-67 expression were nearly collinear with low Ki-67 expression, and the median survival times were similar (Figure 6(d)). Such results imply that patients with high Ki-67 expression may be more sensitive to postoperative adjuvant therapy. By reviewing the literature, we found that Grabowski et al. evaluated chemotherapy responsiveness in patients with low-grade serous ovarian cancer receiving neoadjuvant chemotherapy and showed that Ki − 67 ≥ 4.0% (OR: 44.1, 95% CI: 2.36-825.17, *P* = 0.011) were associated with significantly higher response rates [40]. Similarly, Zhao et al. found that for patients with hepatocellular carcinoma and high expression of Ki-67 (Ki − 67 ≥ 20%), adjuvant hepatic arterial chemoembolization after radical liver tumour resection effectively reduced the probability of postoperative tumour recurrence and prolonged patients' OS; high expression of Ki-67 in postoperative follow-up assessment of patients with hepatocellular carcinoma is an indicator of adjuvant TACE therapy [41]. Recent studies have shown that quiescent cancer cells resist anticancer treatments and underlie cancer recurrence and metastasis. La et al. analyzed quiescent cancer cells and found that low Ki-67 is involved in regulating cancer cell quiescence [42]. In conclusion, previous studies are consistent with our results that the Ki-67 index is a suitable biomarker to evaluate postoperative treatment of ESCC. It can be seen from the EGFR survival curve that both patients with high and low EGFR expressions can benefit from treatment and have more prolonged median survival than untreated patients. From this point of view, EGFR may not be an ideal indicator to assess the need for treatment in ESCC patients. At the same time, we also found that although the patients with high EGFR expression who received treatment had a short-term survival benefit, the survival rate decreased significantly after two years (Figure 7(c)). We speculate that this may be related to the drug resistance caused by high EGFR expression. Patients may require combination therapy with EGFR-targeting drugs (gefitinib or erlotinib).

In this study, we explored the relationship between GPNMB, EGFR, p-PI3K, and Ki-67 in ESCC for the first time and preliminarily confirmed the roles of GPNMB, EGFR, p-PI3K, and Ki-67 in ESCC and their impact on prognosis. However, our study still has certain limitations. First, the consistency of postoperative treatment among patients is not well represented. Currently, postoperative adjuvant therapy for EC patients mainly combines radiotherapy and chemotherapy. However, according to the follow-up results, most patients did not receive the treatment recommended by the guidelines after surgery, which may be related to the nutritional status of patients after esophagectomy and some complications. In addition, patients who received postoperative treatment had treatment imbalances, and some patients could not tolerate chemotherapy and did not complete the established treatment plan. Also, the number of cases in our study was insufficient to explain all statistical issues, so future studies may require a larger sample size. Finally, we are satisfied with the results of the study. Although IHC analysis could not identify the molecular mechanism of action, it did provide a basis for follow-up studies. In the future, further combined cell and animal model experiments are needed to reveal the underlying mechanisms.

## Figures and Tables

**Table 1:** General characteristics of ESCC patients.

Characteristics	Number (n =226)
Age (years, median [range])	60.5 [32-83]
>60	144 (63.7%)
≤60	82 (36.3%)
Tumour size (cm, median [range])	2.5 [0.3-7.5]
<3	68 (30.1%)
≥3	158 (69.9%)
Gender	
Male	161 (71.2%)
Female	65 (28.8%)
Nationality	
Han	115 (50.9%)
Kazak	111 (49.1%)
Location	
Upper	12 (5.3%)
Midpiece	132 (58.4%)
Lower	82 (36.3%)
Differentiation	
Well	59 (26.1%)
Moderate	119 (52.7%)
Poorly	48 (21.2%)
AJCC	
Stage I	17 (7.5%)
Stage II	141 (62.4%)
Stage III	47 (20.8%)
Stage IV	21 (9.3%)
Lymph metastasis	
Negative	150 (66.4%)
Positive	76 (33.6%)
Vessel invasion	
Negative	183 (81.0%)
Positive	43 (19.0%)
Nerve invasion	
Negative	178 (78.8%)
Positive	48 (21.2%)
Treatment	
Treated	148 (65.5%)
Untreated	78 (34.5%)
GPNMB (score range)	0-288
Median	114
EGFR (score range)	0-284
Median	84.5
p-PI3K (score range)	0-276
Median	94.5
Ki-67 (score range)	1-99
Median	67

Abbreviations: ESCC: esophageal squamous cell carcinoma; AJCC: American Joint Committee on Cancer.

**Table 2:** Univariate analysis of factors associated with OS in ESCC patients.

Variable	HR	95.0% CI	*P* value
GPNMB	1.335	0.992-1.797	0.056
EGFR	1.116	0.830-1.501	0.466
p-PI3K	1.619	1.200-2.183	**<**0.001
Ki-67	1.689	1.253-2.276	**<**0.001
Tumour size	1.292	0.930-1.795	0.127
Lymphatic metastasis	1.817	1.338-2.469	**<**0.001
Location	1.041	0.806-1.344	0.760
Differentiated	0.628	0.505-0.781	**<**0.001
Infiltration depth	1.170	0.882-1.551	0.275
Nerve invasion	1.671	1.183-2.360	0.004
Vascular invasion	1.412	0.981-2.032	0.064
Treatment	0.686	0.502-0.938	0.018
Age	1.022	0.753-1.389	0.887
Gender	0.748	0.531-1.053	0.096
Nationality	1.012	0.752-1.362	0.938
AJCC	1.798	1.312-2.463	**<**0.001

Abbreviations: ESCC: esophageal squamous cell carcinoma; OS: overall survival; HR: hazard ratio; CI: confidence interval; AJCC: American Joint Committee on Cancer.

## Data Availability

RNA-seq expression data of 81 patients with ESCC were collected from The Cancer Genome Atlas (TCGA) database (https://portal.gdc.cancer.gov/). Another independent ESCC dataset GSE161533 with 28 matched ESCC samples and normal esophageal samples was obtained from the Gene Expression Omnibus (GEO) database (https://www.ncbi.nlm.nih.gov/geo/query/acc.cgi?acc=GSE161533).

## Supplementary Materials

Supplementary Materials Supplementary Figure: expression of GPNMB, EGFR, p-PI3K, and Ki-67 in normal squamous epithelium. (A, B) GPNMB is expressed in the basal layer in normal esophageal squamous epithelium (A: high magnification; B, low magnification). (C, D) EGFR is expressed in the cell membrane and cytoplasm of normal esophageal squamous epithelium, and the expression level is very low (A, high magnification; B, low magnification). (E, F) p-PI3K is expressed in the nucleus and cytoplasm in normal esophageal squamous epithelium (A, high magnification; B, low magnification). Ki-67 is expressed in the nucleus in normal esophageal squamous epithelium (A, high magnification; B, low magnification). The red line marks the junction between the base of the squamous cell carcinoma and the stroma. Red arrows indicate staining of cancer cells; green arrows indicate staining of mesenchymal cells.
