# Supplementary figures and images for: Prognostic Value of GPNMB, EGFR, p-PI3K, and Ki-67 in Patients with Esophageal Squamous Cell Carcinoma
Source: Anal Cell Pathol (Amst). 2022 Aug 31;2022:9303081. doi: 10.1155/2022/9303081 (PMC9452951; doi:10.1155/2022/9303081)

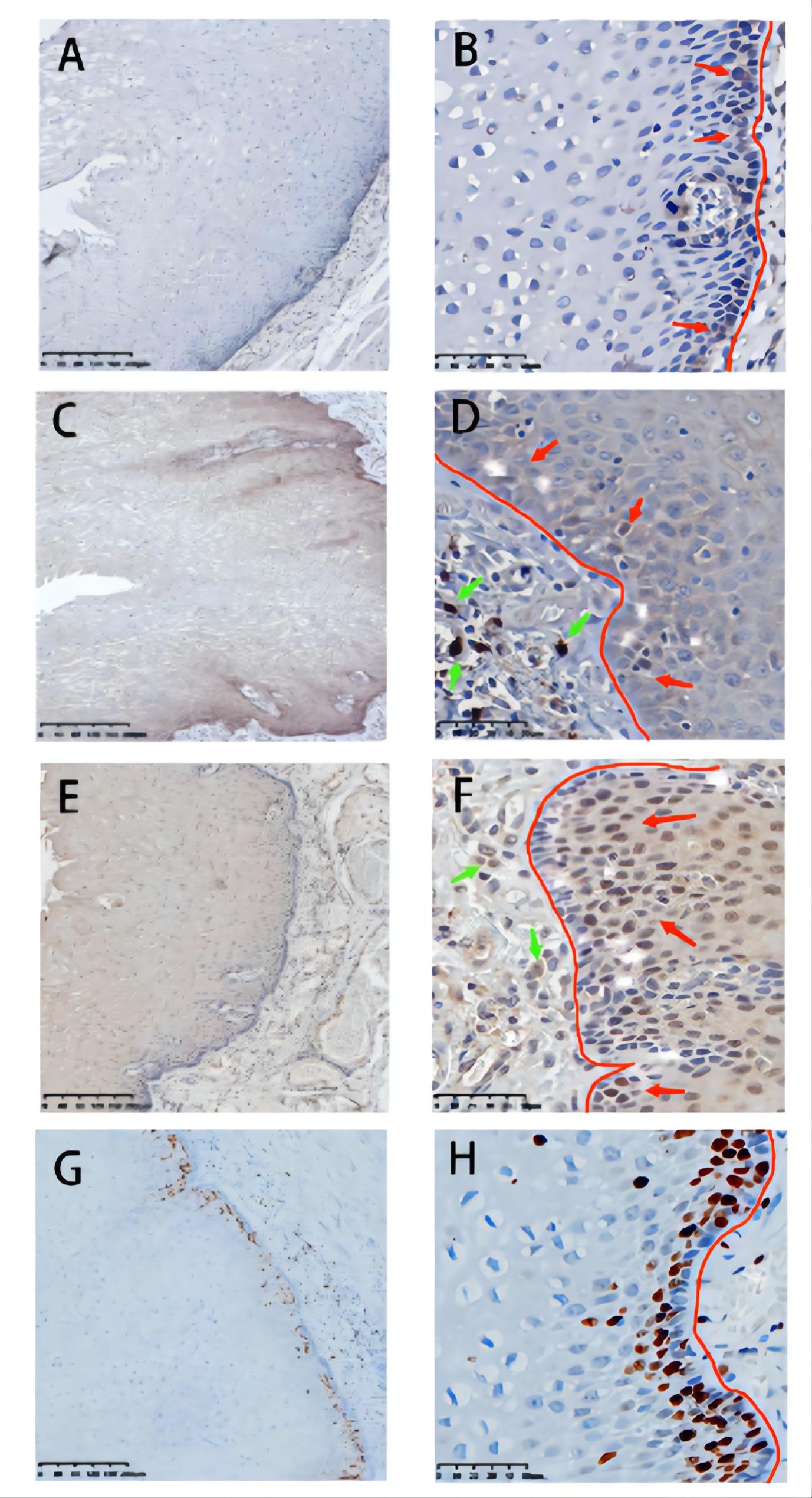

Supplement: Supplementary Materials — Supplementary Figure: expression of GPNMB, EGFR, p-PI3K, and Ki-67 in normal squamous epithelium. (A, B) GPNMB is expressed in the basal layer in normal esophageal squamous epithelium (A: high magnification; B, low magnification). (C, D) EGFR is expressed in the cell membrane and cytoplasm of normal esophageal squamous epithelium, and the expression level is very low (A, high magnification; B, low magnification). (E, F) p-PI3K is expressed in the nucleus and cytoplasm in normal esophageal squamous epithelium (A, high magnification; B, low magnification). Ki-67 is expressed in the nucleus in normal esophageal squamous epithelium (A, high magnification; B, low magnification). The red line marks the junction between the base of the squamous cell carcinoma and the stroma. Red arrows indicate staining of cancer cells; green arrows indicate staining of mesenchymal cells. [file 9303081.f1.jpg]
